# The value of preoperative diagnostic tests in acute appendicitis, retrospective analysis of 196 patients

**DOI:** 10.1186/1749-7922-5-5

**Published:** 2010-02-11

**Authors:** Kemal Memisoglu, Bora Karip, Metin Mestan, Ender Onur

**Affiliations:** 1 General Surgery Department, Fatih Sultan Mehmet Training and Research Hospital, E-5, Bostanci, Istanbul, Turkey

## Abstract

**Background:**

In this study our aim was to evaluate the diagnostic value of preoperative laboratory and radiological studies for appendicitis.

**Methods:**

The clinical data of 196 patients who have undergone conventional appendectomy between March 2007 and April 2008 were collected retrospectively. Patients were examined for age, sex, white blood cell count, ultrasonography results, histopathological diagnosis and hospital stay.

**Results:**

Negative appendectomy rate was 17.3% (27% for female, 11.5% for male). White blood cell counts were found to be high in 83% for acute appendicitis group and %61 for negative appendectomy group. There were 66 (34%) patients who had negative USG findings for acute appendicitis. Of these patients, histopathological examination revealed acute appendicitis in 46 patients whereas 20 patients had normal appendix. Hospital stays were 2.79 +/- 1.9 and 2.66 +/- 1.7 days for negative and positive appendicectomies respectively.

**Conclusions:**

Besides the improvement of diagnostic tests for acute appendicitis, we could not sufficiently reduce the negative appendectomy rate.

## Background

Appendicectomy is still the most common procedure in general surgery practice but diagnostic failure may still occur and this leads to delay in treatment or negative (non-therapeutic) appendectomies. We aimed to analyze retrospectively the diagnostic efficiency of the preoperative tests in relation with histopathologic results.

## Methods

Data of the 277 conventional appendectomies performed for acute appendicitis (AA) between March 2007 and April 2008 were collected. Fifteen patients with perforated appendicitis, 23 patients whose preoperative laboratory tests performed at another centre and 43 patients operated on without preoperative ultrasonography (USG) were excluded. In the remaining 196 patients, all had clinical findings such as, history of anorexia, pain followed by nausea, right lower quadrant pain, vomiting, rebound tenderness, guarding, rigidity and conventional appendectomies were carried out. A radiologist performed a graded compression USG (Siemens Sonoline G50) with a 3.5 MHz convex and 7.5 MHz linear probe. Data for age, sex, white blood cell count, abdominal USG results, histological findings and hospital stay were collected. White blood cell count, higher than 10500/mm^3 ^was accepted as leukocytosis. Primary criterion for diagnosing AA by USG was the evidence of a non-compressible appendix and a measured diameter of greater than 7 mm. Other supporting criteria were echogenic periappendiceal mesenteric/omental fat, peri-appendiceal fluid collection and mesenteric lymphadenopathy. USG results including one of these were added positive USG for AA group. Criteria of histological acute appendicitis accepted as infiltration of the muscularis propria with polymorphonuclear cells. Pathology results as -appendix vermicularis- without any additional finding were accepted as negative appendectomy (NA). White blood cell counts, USG findings, hospital stay were compared between AA and NA group. All statistical analysis were performed using SPSS for Windows (version 15·0). *P*-values less than 0.05 were accepted as significant.

## Results

In this study we presented 122 male (62.2%) and 74 female (37.8%) patients with median 27 years old (range 7-81 years) respectively. White blood cell counts were found to be high (>10500/mm^3^) in 80% while it was 83% for AA group and %61 for NA group (p > 0.05). There were 66 (34%) patients who had no USG findings for acute appendicitis. Of these, 46 (70%) patients were observed to have histologically proved AA. There were 130 patients who had positive USG findings for AA and 11% of these had histologically normal appendix.

**Figure 1:**
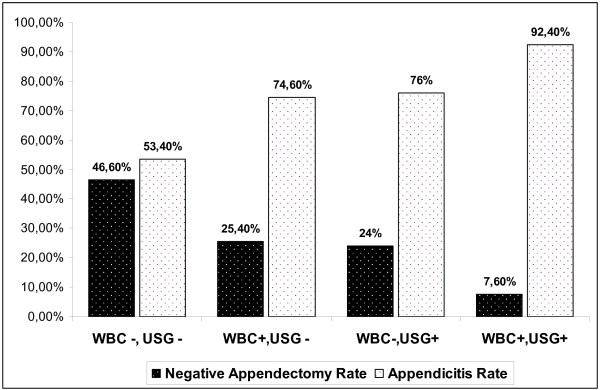
**Percentage of negative appendicectomies and appendicitis through the patients due to WBC levels and USG findings**.

Negative appendectomy rate (NAR) was 17.3%; this rate was 11.5% for male and %27 for female patients (p = 0,003) (Table 1). Negative appendectomy rate (NAR) decreased to 7,6% when white blood cell count was high and USG findings were confirming appendicitis, whereas NAR was 46% in the patients who had normal white blood cell counts and normal USG findings (Figure 1).

**Table 1:** Negative appendicectomy rates

	HISTOPATHOLOGY		
	Negative	Positive	Total
**Male**	14 (11.5%)	108 (88.5%)	122 (62.2%)
**Female**	20 (27%)	54 (73%)	74 (37.8%)
			
**Total**	34 (17.3%)	162 (82.7%)	196 (100%)

Ultrasonography had a sensitivity of 71.6% and a specificity of 58%. The predictive value of a positive test was 89% and the predictive value of a negative test was 30%.

There was no statistically significant difference between the length of postoperative hospital stay for acute appendicitis and negative appendectomy group (2.79 +/- 1.9 and 2.66 +/- 1.7 days, p > 0.05)

## Discussion

Appendicitis is a very common disease with a lifetime occurrence of 7 percent [1]. Main symptom is right lower quadrant pain with anorexia and vomiting. Routine examination of a suspicious acute appendicitis patient consists complete blood count and urinalysis. The most important diagnostic tool is still physical examination but recently use of imaging studies is increasing day by day. This is a result of the need for early diagnose and treatment to achieve less perforation rate and complication [2].

In this study all 196 patients were demonstrating positive symptoms and physical signs for appendicitis. White blood cell counts were high for the 80% of the patients. Elangovan et al found high levels of white blood cell count in AA patients 80 percent [3]. Unfortunately, the white blood cell is elevated in up to 70 percent of patients with other causes of right lower quadrant pain [4]. NAR were 13.4% and 32.5% in the patients who had high and normal white blood cell counts, respectively.

We found our NAR as 17.3%. Kyuseok et al studied 339 patients in two groups as preoperative no imaging and imaging studies and they found their NAR as 20.6 percent and 6.6 percent [5]. Hassan et al found, being younger than 21 years old, female gender, lower levels of polymorphonuclear leukocyt and lower heart rates as a risk factor for negative appendectomy [6]. Singhal et al showed 18.2 percent NAR for males and 48.2 for females at their study group [7]. Our NAR was 11.5 percent for male patients and 27 percent for females.

Radiology with the help of improving technology gets more space in the diagnose and differential diagnose for acute abdomen patients. We used USG for 196 suspicious acute appendicitis patient and found ultrasonography had a sensitivity of 71.6% and a specificity of 58%. The predictive value of a positive test was 89% and the predictive value of a negative test was 30%. Rajeev gave this ratios at his study on 118 preoperatively USG performed appendectomy patients as 63.3%, 82.14%, 91.93% and 41.07% [8]. Another study comparing 200 USG negative patients to 200 USG positive, NAR was found 4.7% for positive group [9]. Suma evaluated 1447 suspicious acute appendicitis patient with USG, 368 (25%) were positive for appendicitis and 7 were false positive. Remaining 1079, 173 patients (12%) had an other diagnose due to USG and 906 patients' complaints regressed during follow up. This study gave a sensitivity and specificity of 98% and 99%. The predictive value of a positive and negative test were 98% and 99% with %99 overall diagnostic accuracy [10]. Difficulties with ultrasonography include identification of normal appendix to rule out acute appendicitis. Visualization of a normal appendix is more difficult in patients with a large body habitus and when there is an associated bowel obstruction, which causes overlying gas-filled loops of bowel. Accuracy of ultrasonography also decreases with retrocecal location of the appendix. Meckel's diverticulum, cecal diverticulitis, inflammatory bowel disease, pelvic inflammatory disease, and endometriosis can cause false-positive ultrasound results. Patients often complain of the pressure during evaluation. As seen above diagnostic value of USG can not be predictable, may be due to the experience of the radiologist, patient factors or technique used.

Today emergency service practitioners are using computerized tomography (CT) for acute abdomen patients more and this may cause reduced rates of NAR. Motoki used CT for AA and published sensitivity and a specificity of 98.9% and 75%, the predictive value of a positive test as 96% and negative test as 90% [11]. Another CT technique uses rectal gastrografin lavmane. Advantages of this technique are, causing no delay for surgery due to oral intake, no need for intravenous contrast and ability to show not only inflamed appendix but also periappendicular inflammatory changes such as mesenteric edema [12,13].

Hannah et al analyzed the imagination studies as a factor of a delay in surgery and could not show any difference between non-imaging group and imaging group except a reduce of NAR from 10% to 3% favoring the latter [14]. Recent studies are showing short delays due to radiologic examinations have no bad effect on outcome for AA patients but they reduce NAR ratios [15,16].

There were no statistically significant difference between the length of primary hospital stay for AA and NA group (2.79 +/- 1.9 and 2.66 +/- 1.7 days, p > 0.05). Kuzma showed no difference between complication rates for AA and NA groups [17]. Differences in the course for these two groups seem to be that NA patients re-admit emergency services more due to their unsolved problem although appendicitis patients meet more septic complications [18].

## Conclusions

The diagnosis of appendicitis remains essentially clinical. Our NAR was 11.5 percent for male patients and 27 percent for females. Despite modern techniques, NA rates are still a problem for surgeons. If there is a doubt about the diagnose although leukocyte levels and ultrasonography results are normal, especially for female patients performing further radiologic examinations such as CT can be favorable.

## Abbreviations

AA: acute appendicitis; NA: negative appendicectomy; NAR: negative appendicectomy rate; USG: ultrasonography; CT: computerized tomography.

## Competing interests

The authors declare that they have no competing interests.

## Authors' contributions

KM and BK designed the study, collected and analyzed data. They drafted the manuscript.

MM and EO helped collecting the data, reviewing literature, statistical analyze and preparation of the manuscript

All authors have read and approved the main manuscript.
